# Comparison of Marginal Bone Loss in Simultaneous Versus Delayed Implant Placement Following Horizontal Ridge Augmentation with Autogenous Lateral Ramus Bone Block

**DOI:** 10.30476/dentjods.2022.92419.1641

**Published:** 2023-06-01

**Authors:** Reza Tabrizi, Hassan Mohajerani, Hamidreza Moslemi, Shervin Shafiei, Shobeir Majdi

**Affiliations:** 1 Dept. of Oral and Maxillofacial Surgery, School of Dentistry, Shahid Beheshti University of Medical Sciences, Tehran, Iran; 2 Postgraduate student Dept. of Oral and Maxillofacial Surgery, School of Dentistry, Shahid Beheshti University of Medical Sciences, Tehran, Iran; 3 Dept. of Oral and Maxillofacial Surgery, School of Dentistry, Qom University of Medical Sciences, Qom, Iran

**Keywords:** Alveolar ridge augmentation, Autogenous bone grafts, Dental implants

## Abstract

**Statement of the Problem:**

Alveolar ridge resorption after tooth extraction may interfere with optimal dental implant placement.

**Purpose:**

This study aimed to compare the marginal bone loss (MBL) and thickness of the buccal aspect of the augmented site in simultaneous versus delayed implant placement following lateral ramus horizontal ridge augmentation in the posterior mandible.

**Materials and Method:**

This prospective cohort study was conducted on patients who required horizontal bone augmentation of the posterior mandible using lateral ramus autogenous bone graft. Patients were divided into two groups of simultaneous implant placement (group 1) and delayed implant placement (group 2). Cone-beam computed tomography (CBCT) images were obtained before augmentation, at the time of implant placement, and 10 months later (6 months after implant loading). MBL and thickness of the buccal aspect were evaluated over time.

**Results:**

There were 18 patients in the group 1 and 16 patients in the group 2. Analysis of the CBCT scans demonstrated that the mean MBL was 1.21±0.35mm in the group 1 and 1.08±0.19mm in the group 2, with no significant difference between the two groups (*p*= 0.19). Thickness of the buccal aspect of the augmented site at the time of implant placement was 1.85±0.20mm in the group 1 and 2.16±0.29 mm in the group 2, with a significant difference (*p*< 0.001). However, data analysis regarding changes in the buccal plate thickness showed no significant difference between the two groups (*p*= 0.36).

**Conclusion:**

According to the results of this study, there was no significant difference in M-BL and post-operative changes in the thickness of the buccal aspect of the augmented sites with onlay lateral ramus bone blocks between simultaneous and delayed implant placement.

## Introduction

Alveolar ridge resorption after tooth extraction may interfere with optimal dental implant placement. The success of osteointegration depends on quality and quantity of bone in the recipient sites. Insufficient bone volume can compromise a proper implant positioning [ 1 ]. Malpositioning of dental implants can affect both the functional and esthetic outcomes [ 2 ]; thus, alveolar reconstruction is a prerequisite for implant placement [ 3 ]. 

A variety of surgical reconstruction options have been recommended for alveolar ridge rehabilitation. Autogenous bone blocks are still the gold standard for both vertical and horizontal augmentation because of their optimal osteoconductivity, osteoinductivity, and osteogenic potential [ 3 - 4 ]. Recent studies showed promising results regarding graft resorption and implant success rate following bone augmentation with intraoral autogenous bone blocks. The mandibular ramus and symphysis are the most popular intraoral sources for harvesting autogenous cortical and/or corticocancellous bone blocks [ 5 - 7 ]. 

Little is known about the effect of immediate and delayed implant placement in the grafted site on the long-term implant success rate and graft resorption [ 8 ]. Knowledge about the resorption rate of the graft and the final volume gain after final remodeling is imperative for treatment planning before implant placement. The primary goal of this study was to compare the marginal bone loss (MBL) and the thickness of the buccal aspect of the augmented site in simultaneous versus delayed implant placement following lateral ramus horizontal ridge augmentation in the posterior mandible.

## Materials and Method

The authors designed a prospective cohort study on patients referred to the Department of Oral and Maxillofacial Surgery of Shahid Beheshti Dental School between October 2017 and December 2019 for dental implant treatment. One surgeon performed all treatments and he was experienced in alveolar ridge reconstruction techniques. The inclusion criteria consisted of patients >18 years of age with good oral hygiene and ASA I and II systemic condition candidates for dental implant placement in the severely atrophic posterior mandible (alveolar ridge width less than 4 mm with an adequate height). Patients with systemic conditions (such as uncontrolled diabetes mellitus), autoimmune disorders, previous chemotherapy, previous head and neck radiotherapy, pregnancy, the need for vertical bone augmentation, poor oral hygiene, active periodontal infection, with a history of bone augmentation procedures at the implant site, patients taking medications affecting bone metabolism, smokers, and those who refused to show up for the recall sessions were excluded. The patients were divided into two groups based on intraoperative evaluations. Group 1 patients underwent simultaneous implant placement since a dental implant could be placed for them with adequate primary stability (>25 Ncm) and proper position and angulation (ridge width between 3 or 4 mm). Group 2 patients underwent delayed dental implant placement (four months after augmentation). Demographic data, including the age and gender of patients, were collected in a data collection form. The aim and design of the study and the surgical procedure were thoroughly explained to patients, and written informed consent was obtained from all participants. The Ethics Committee of Shahid Beheshti University of Medical Sciences approved the protocol of this study (code: IR.SBMU.DRC.REC.1398.061). In addition, this study was performed according to the principles outlined by the World Medical Association's *Declaration of Helsinki* on experiments involving human subjects, as revised in 2000. 

All patients underwent complete oral soft and hard tissue evaluation. The width of the implant site and ridge angulation were assessed using cone-beam computed tomography (CBCT) before the graft harvesting to determine the size of bone blocks required for horizontal augmentation. 

### Bone graft harvesting

All patients received prophylactic antibiotics (2 g amoxicillin or 600 mg clindamycin if allergic to penicillin) 1 hour before surgery and rinsed their mouth with 0.2% chlorhexidine mouthwash. Local anesthesia was induced using 2% lidocaine with 1:100,000 epinephrine (Darupakhsh; Tehran, Iran) injected at the donor site. A mucosal incision was made to access the lateral ramus area starting from the concavity formed between the border of the ascending ramus and the external oblique ridge and extending towards the buccal aspect of the second molar medial to the external oblique ridge. A full-thickness flap was elevated, exposing the lateral aspect of the ramus. The osteotomy was carried out using a piezosurgery unit (VarioSurg; NSK, Japan). The outer cortical plate was split based on the required size and detached by a thin chisel from the donor site. The flap was sutured with 4-0 Vicryl sutures (Ethicon Inc., Johnson & Johnson Company, Somerville, NJ, USA). 

### Fixation of the bone block at the recipient site

**Figure 1:**
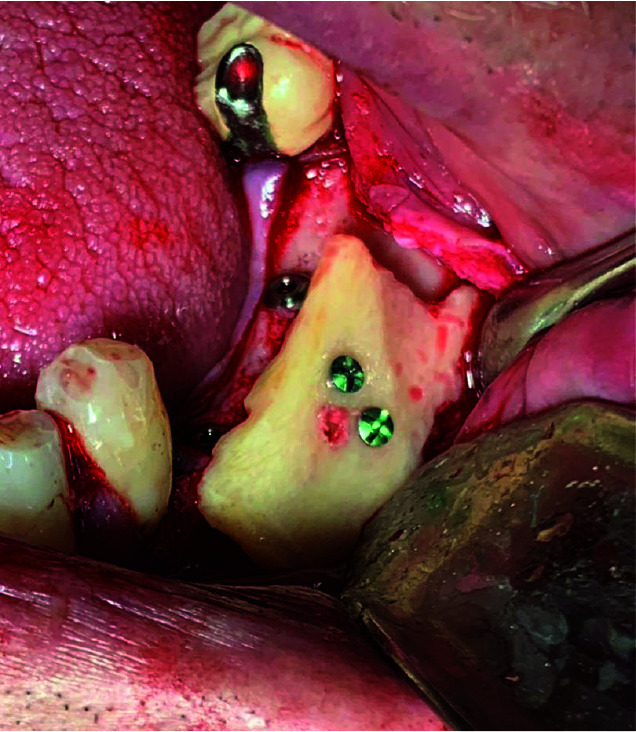
Clinical view of implant site after fixation of lateral ramus block bone graft

**Figure 2:**
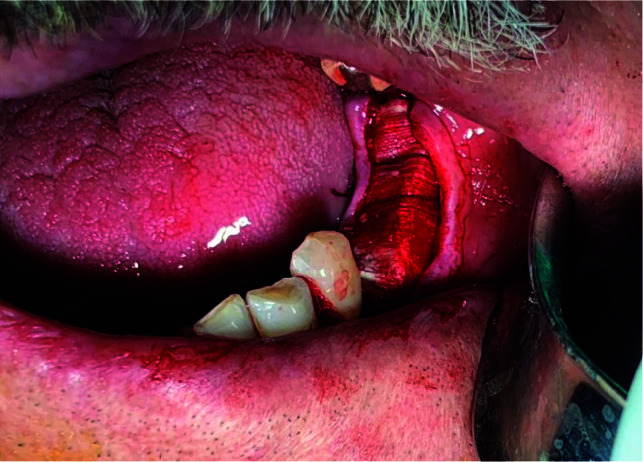
Clinical view of implant site after membrane placement

Local anesthesia was administered at the recipient site, and a crestal mucosal incision was made in the edentulous area. The mesial and distal aspects of the crestal incision were connected to two releasing incisions, and a mucoperiosteal flap was elevated. The cortical bone at the recipient site was perforated using drills under saline irrigation to ensure vascularization of the bone block. The bone block was re-contoured using a diamond bur and adapted to the recipient site. It was fixed to the residual ridge with fixation miniscrews (Jeil, Seoul, Republic of Korea). The fixation of the bone block was performed after implant placement in the group 1 (Figure 1). The gaps between the recipient site and the bone blocks were filled with bone substitute material (Cerab-one®), and a membrane (Jason®) covered the grafting site (Figure 2). Tension-free closure of the flap was performed with interrupted sutures. The patients were instructed to use postoperative antibiotics (amoxicillin 500 mg/8 h or clindamycin 300 mg/8 h if allergic to penicillin) and 0.2% chlorhexidine mouthwash for seven days.

### Dental implant placement

In the group 1, dental implants were placed at the time of bone grafting, while, in the group 2, dental implants were placed four months after bone grafting. At the time of implant placement, a crestal incision was made with two short releasing incisions in the mesial and distal aspects. Miniscrews, which could interfere with the path of implant fixtures, were removed. The implants had 3.5 or 4mm diameter and 10 mm length (Osstem® fixture, TS III SA). All implants were placed with good primary stability. Patients were instructed to use 0.2% chlorhexidine mouthwash for seven days after implant placement.

### Implants loading

Metal-ceramic crowns were fabricated and loaded for patients four months after implant placement. Optimal occlusion was carefully assessed on an articulator and intraorally for all restorations. The crowns were cemented with temporary cement (Temp-Bond ®; Kerr, Orange, CA, USA). 

### Clinical evaluation

Patients in both groups were evaluated for bone grafting complications such as graft or block exposure, infection, mobilization of the bone block, or loss of bone particles. The recall sessions also evaluated the presence/absence of pain, sensitivity, suppuration, or exudation at the implant site and implant mobility. 

### Radiographic evaluation

**Figure 3:**
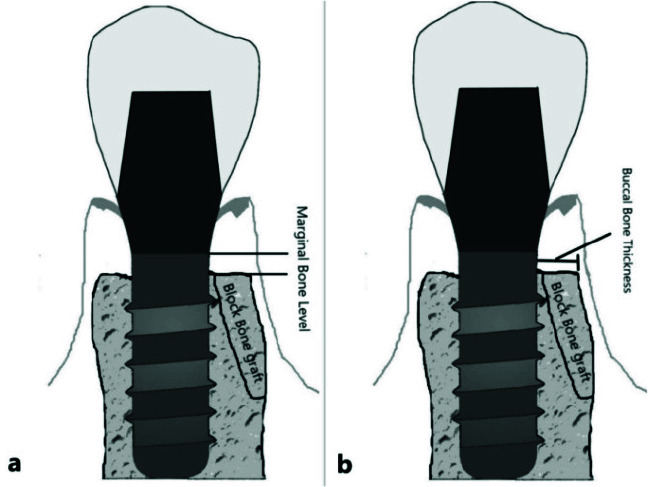
**a:** Measuring marginal bone level (MBL) in CBCT images, **b:** Measuring buccal bone thickness (BBT) in CBCT images

The authors performed radiographic evaluation based on CBCT images taken immediately after implant placement and ten months later (6 months after implant loading). The reproducibility of measurements made at different time points was certified by using anatomical landmarks and screws as reference points. All CBCT scans were taken using NewTom VGi CBCT scanner (QR SRL Company, Verona, Italy) and analyzed with NewTom NNT Viewer version 5.3 software (Quantitative Radiology, Verona, Italy). The CBCT scans were obtained with an 8×12 cm field of view and 200-µm voxel size. Analysis was carried out by an experienced oral and maxillofacial radiologist. To assess the MBL, the changes in marginal bone level were recorded between the two time points. The marginal bone level was considered the distance between the implant platform and the alveolar bone crest in vertical dimension at the mesial and distal surfaces of each implant and was calculated based on the mesial and distal measurements made on each image (Figure 3a). The buccal bone thickness (BBT) at the buccal aspect of the augmented site was also evaluated by measuring the distance between the outer surface of the implant fixture and the most lateral border of the augmented bone (Figure 3b). It was measured at the time of implant placement (BBT1), and its changes over time were also evaluated according to the baseline measurements (BBT-changes).

### Statistical analysis

The statistical analyses were performed using SPSS version 23 (SPSS Inc., IL, USA). The independent t-test was used to compare the mean age, MBL, BBT1, and BBT-changes over time between the two groups. The Chi-square test was applied to compare gender distribution. p Values <0.05 were considered statistically significant.

## Results

A total of 34 patients (18 patients in the group1 and 16 patients in the group 2) participated in this study. Two patients in the group 2 were excluded as they refused to show up for the follow-up sessions. Table 1 presents the demographic information of patients. There were no significant differences between the two groups in patients' mean age or gender distribution (*p*= 0.43 and *p*= 0.48, respectively). 

**Table 1:** Demographic information of patients in simultaneous implant placement and delayed implant placement groups

	Age			Gender (N)		
	Mean	SD	*P* value	Male	Female	*p* value
Group 1	37.44	7.60	0.44	8	10	0.68
Group 2	39.56	8.13		6	10	

Group 1=Simultaneous implant placement, Group 2= Delayed implant placement, SD=Standard deviation

Graft or block exposure, infection, mobilization of the bone block, or loss of bone particles were not observed in any patient. None of the implants in the two groups showed pain, sensitivity, suppuration, exudation, clinically detectable implant mobility, or peri-implant radiolucency. Donor site showed appropriate healing with no uncommon complications.

MBL was evaluated in the two groups ten months after implant placement. CBCT analysis demonstrated that the mean MBL was 1.21±0.35mm in the group1 and 1.08±0.19mm in the group 2. Statistical analysis showed no significant difference between the two groups in this respect (*p*= 0.19). The thickness of the buccal aspect of the augmented site was also evaluated. The mean BBT1 was 1.85±0.20mm in the group1 and 2.16±0.29mm in the group 2. A significant difference was observed in BBT1 between the two groups (*p*< 0.001). However, data analysis regarding BBT changes showed no significant difference between the two groups. BBT-changes were 0.71±0.19 mm in the group1 and 0.66±0.14 mm in the group 2 (*p*= 0.36) (Table 2).

**Table 2:** Marginal bone loss and thickness of the buccal aspect based on cone-beam computed tomography scans in simultaneous implant placement and delayed implant placement groups

	Group 1		Group 2		*P* value
	Mean	SD	Mean	SD	
MBL	1.21	0.35	1.08	0.19	0.19
BBT1	1.85	0.20	2.16	0.29	0.001*
BBT-changes	0.71	0.19	0.66	0.14	0.36

Group 1=Simultaneous implant placement, Group 2= Delayed implant placement, MBL=Marginal bone loss, BBT1= Thickness of the buccal aspect of the augmented site at the time of implant placement, BBT-Changes= Changes in thickness of the buccal aspect of the augmented site at the time of implant placement and 10 months later, SD=Standard deviation, *=Significant difference

## Discussion

This study was designed to compare MBL and thickness of the buccal aspect of the augmented site in simultaneous versus delayed implant placement following lateral ramus horizontal ridge augmentation in the posterior mandible. Our findings suggested that the one-stage protocol (lateral augmentation with simultaneous implant placement) could be performed when adequate bone volume is available for favorable primary stability and position of implants. Moreover, the results revealed promising outcomes regarding the rate and final amount of graft resorption, which are important to guarantee the long-term implant stability. 

In our study, the mean of MBL in one stage and two stage protocols was not different. Therefore, one-stage protocol has an advantage to two-stage protocol in reducing the treatment time and the number of surgical process. Survival and success rates of dental implants placed in horizontally resorbed edentulous ridges, which are augmented with block bone grafts, are similar to those of implants in native bone [ 9 ].

The two-stage protocol has also been suggested for some circumstances, for example, when correct three-dimensional position of implant and its final restoration cannot be achieved in the first surgical appointment or when confronting a severe horizontally atrophic ridge with one-wall morphology. Several studies reported acceptable results for the staged procedure in large horizontal defects reconstructed by lateral ramus or symphysis block grafts [ 4 , 7 - 9 ]. However, the most prominent benefits of the one-stage protocol such as one surgical session especially in medically compromised patients and early loading with final restoration will be lost in the two-stage protocol.

In terms of bone volume regain and MBL around dental implants in long-term, the onlay bone grafting of horizontal defects has shown less bone resorption during the follow-up period compared with guided bone regeneration. However, most relevant studies reported similar results regarding implant survival and implant insertion torque between the guided bone regeneration and onlay bone grafting using intraoral donor sites [ 4 , 6 - 7 , 10 ]. Recent studies regarding bone volume resorption after lateral augmentation with lateral ramus grafts (two-stage protocol) mostly reported a clinically acceptable range. Gultekin *et al*. [ 7 ] reported 7.2% volume loss after 6 months of follow-up following ramus block bone grafting. Chappuis *et al*. [ 11 ] also reported only 7.7% (0.38 mm) surface resorption after 10 years in horizontally grafted sites with autogenous bone blocks. In a study by Cordaro *et al*. [ 12 ], the mean lateral augmentation gain immediately after augmentation with bone blocks was 6.5mm, which decreased to 5mm during the healing period. The immediate horizontal width gain after block grafts was variable with a noticeable range among different studies, mainly because of different surgical approaches. However, the resorption rates during the postoperative phase were clinically acceptable in all the above-mentioned studies [ 5 - 6 ]. The buccal bone plate thickness in two-stage protocol was more than one stage protocol immediately after implant placement (2.16±0.29mm and 1.85±0.20mm, respectively).

The thicker buccal bone plate in two-stage protocol could be due to sufficient bone thickness during implant placement, which allows surgeons to determine an appropriate position for implant insertion. The buccal bone resorption rate was not different in one stage and two stage protocols in our study (0.71±0.19 and 0.66±0.14, respectively); the rate of buccal bone resorption was 38.37% of the primary thickness of the buccal plate in one-stage protocol and 30.55 % in two-stage protocol. It could be concluded that an average of 30% resorption of the primary thickness of the buccal plate could be expected whether the one-stage or two-stage protocol is conducted for the horizontal ridge augmentation.

Rasmusson *et al*. [ 13] evaluated the effect of simultaneous implant placement on the stability of titanium implants placed in onlay bone grafts in the skull of rabbits. The resonance frequency analysis demonstrated higher implant-bone contact in implants inserted 8 weeks after bone grafting (delayed protocol); however, the differences were not significant. In a study similar to our study by Peñarrocha-Diago *et al*. [ 8 ] the patients underwent lateral ridge augmentation by intraoral block grafts (lateral ramus and symphysis donor sites), and MBL and implant survival rate were evaluated. The implant success rate was 89.5% and 96.9% in simultaneous and delayed implant placement protocols, respectively. One year after implant loading, the MBL was significantly greater in the simultaneous implant placement protocol (0.69mm in the simultaneous and 0.20 mm in the delayed protocol), which was in contrast to our results. Other reports showed acceptable results following simultaneous placement of implants in horizontally grafted areas [ 14 - 15 ]. For example, Boronat *et al*. [ 14 ] in their retrospective study on 37 patients who underwent simultaneous implant placement after lateral augmentation with bone block grafts reported implant success rate of about 95%, and a mean bone loss of 0.64 mm after one year. Further studies with larger sample size are required on simultaneous implant placement after lateral augmentation for more reliable treatment planning in patients who are suitable candidates for this technique based on their specific defect morphology and the remaining bone volume. 

## Conclusion

According to the results of this study, there was no significant difference in MBL and post-operative changes in the thickness of the buccal aspect of the augmented sites with onlay lateral ramus bone blocks between simultaneous and delayed implant placement. 

## Conflicts of Interest

The authors declare that they have no conflict of interest.
